# Optical Fiber Geometry: Accurate Measurement of Cladding Diameter

**DOI:** 10.6028/jres.098.015

**Published:** 1993

**Authors:** Matt Young, Paul D. Hale, Steven E. Mechels

**Affiliations:** National Institute of Standards and Technology, Boulder, CO 80303-3328

**Keywords:** critical dimensional measurements, gray scale measurement, interference microscope, micrometer, microscope, optical fiber, optical fiber geometry, scanning confocal microscope, Standard Reference Material, video microscope

## Abstract

We have developed three instruments for accurate measurement of optieal fiber cladding diameter: a contact micrometer, a scanning confocal microscope, and a white-light interference microscope. Each instrument has an estimated uncertainty (3 standard deviations) of 50 nm or less, but the confocal microscope may display a 20 nm systematic error as well. The micrometer is used to generate Standard Reference Materials that are commercially available.

## 1. Introduction

This paper reports the development of an artifact standard (a Standard Reference Material, or SRM) for video microscopes devoted to measuring optical fiber geometry [1]. Specifically, we have developed three devices, a contact micrometer [2], a scanning confocal microscope [3,4], and a white-light interference microscope [5], that are capable of absolute measurements with accuracy between 50 and 100 nm. Much of this material has been reported in various forums while it was in progress; this paper summarizes.

A video microscope dedicated to fiber geometry is called a gray scale system by the Telecommunications Industry Association (TIA) [6]. Gray scale systems are typically used to determine the outer, or cladding, diameter of a cleaved fiber end; the noncircularity of the cladding; and the decentering, or concentricity error, between the core and the cladding. Measurements of noncircularity and de-centering do not require high absolute accuracy. Cladding diameter, by contrast, must be measured within 0.1 µm or less if we are to provide standards for the manufacture of efficient connectors that do not require manual adjustment.

Measurements made with video microscopes, unfortunately, may well suffer from a systematic error of a few-tenths micrometer [7]. The TIA subcommittee we work with wanted a transfer standard so that they could correct for this systematic error. It was, however, reluctant to accept an artifact standard other than an optical fiber because the measured result is a function of illumination and also because reflection from a metal film displays phase shifts that are not present in reflection from a glass edge. Indeed, the concern about phase shifts is not misplaced: we have measured widths of chromium-on-glass lines with a scanning confocal microscope and found the measured results to change by nearly 0.1 µm with polarization [4]. At any rate, even if a chromium-on-glass standard had been adopted, it would have been necessary to measure a fiber very accurately to verify the relevance of the chromium standard. We therefore undertook to develop a fiber artifact standard for calibrating the gray scale systems. The National Physical Laboratory in the U. K. has a similar program [8].

In this paper, we will describe the contact micrometer, scanning confocal microscope, and white-light interference microscope that we have set up with the goal of measuring the cladding diameter within 0.1 µm or less. Because of the ease of using the micrometer, it is the instrument that we use regularly to prepare standards. Because of the need for accuracy (as opposed to precision or repeatability), however, we require the other methods to verify the accuracy of the micrometer.

## 2. Contact Micrometer

**Fig. 1:**
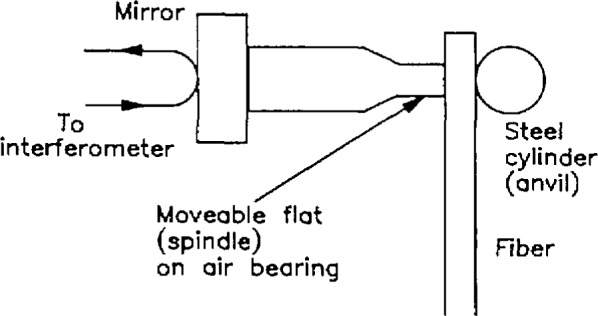
A schematic drawing of the contact micrometer. The moveable spindle is pressed against the fiber with a known force, and the position of the spindle is monitored interferometrically.

We acquired a contact micrometer from our colleague Theodore Doiron of the Precision Engineering Division in Gaithersburg, Maryland. The *anvil*, or stationary part, is a steel post about 3.8 mm in diameter (Fig. 1). The *spindle*, or moving part, rides horizontally on an air bearing and is pressed against the anvil with a known force; this force is developed by hanging a weight (not shown) over a pulley. The end of the spindle is a cylinder about 5 mm in diameter. A mirror is contacted to the opposite end of the spindle, and its position is measured by a commercial interferometer that has a least count of 1.25 nm. We estimate its accuracy to be a few nanometers owing to changes in barometric pressure, temperature, and humidity; cosine error; and so on. The reflecting surface of the mirror is perpendicular to the axis of the spindle, within less than 5′.

### 2.1 Optical Polishing

We had both the spindle and the anvil polished with hard laps in an optical shop. The spindle must be flat, and the anvil must be cylindrical or conical, not barrel- or hourglass-shaped, or systematic errors will arise.

Measurements are performed by first pressing the fiber between the spindle and the anvil and using the interferometer to measure the position of the spindle. Then, the fiber is removed, and the spindle is brought into contact with the anvil. The difference between the two positions is the diameter of the fiber, apart from a correction for compression (below).

The anvil is epoxied under pressure to a vertical vee groove. The spindle and the end of the anvil may be made parallel by inserting a fiber and measuring its diameter both above and below the center of the spindle. The vee groove that holds the anvil may be shimmed for coarse adjustment. For fine adjustment, the end of the spindle has been polished flat but at an angle of a few minutes. The spindle may be rotated incrementally until the measurements above and below the center agree within a few nanometers.

A major problem is to ensure that there is no burr, or defect, projecting from the anvil. If there is such a defect, the micrometer will not close properly, and the measurements will be low, except rarely when the fiber seats on top of the burr. It is helpful, therefore, to have advance knowledge of the diameter of the specimen (apart from the correction for compression, below). This may be obtained by deliberately misaligning the spindle. Then, there is only one point of contact between the spindle and the anvil. (This point is comparatively hard to find, so it is not practical to design a micrometer in which the spindle and the anvil are not parallel.) With the spindle misaligned, we measure the diameter of the fiber at the point of contact. Next, we align the micrometer so that the spindle and the anvil are parallel as outlined in the previous paragraph. If the anvil is free of burrs or contamination, the measured diameter is about the same as the diameter obtained when the parts were misaligned. In addition, the measurements above the center of the spindle and those below converge (as the spindle is rotated) to an unreasonably small value when there is a burr on the anvil. Thus, there are two clues that indicate the presence or absence of a burr; it is necessary to perform a great many alignments of the spindle, preferably at different locations on the anvil, to satisfy yourself that the micrometer is accurate. We estimate that the error due to the presence of a burr is not larger than 10 nm.

**Fig. 2:**
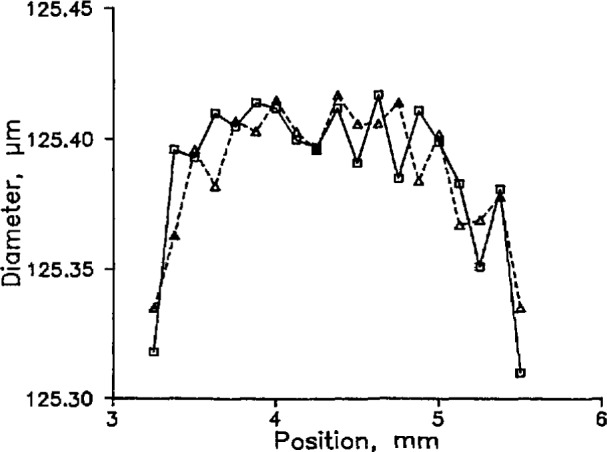
Two consecutive sets of measurements of the diameter of the fiber as a function of position along the anvil.

To test the accuracy and parallelism of the surfaces, we next inserted a fiber and measured its apparent diameter at various points along the line of contact between the spindle and the anvil. Figure 2 shows the result. In the center of the plot, where the spindle is fairly flat, the total scatter of the data is about 32 nm and is partly the result of electronic noise and vibration but mostly the result of roughness in the surfaces. We presume, however, that the “correct” answer is the largest value, because the highest point on either surface is what actually makes contact when the fiber is removed. When the fiber is positioned anywhere else, the spindle moves less than one full fiber diameter when the second measurement is made. If we took, say, six measurements at different positions, the most probable result would be the mean of the relevant points in Fig. 2. But the presumed correct result is about 16 nm larger than this mean, so we add 16 nm to the measured value and assign an uncertainty equal to half that, or 8 nm.

### 2.2 Compression

The fiber and the anvil make a point contact, so there is measurable compression there. The compression depends on the elastic constants of the materials and can in principle be calculated from formulas developed by Puttock and Thwaite [9]. The spindle contacts the fiber in a line, not a point; the calculated compression is only a few nanometers. Similarly, when the fiber is removed, the spindle contacts the anvil in a line; the corresponding compression is also a few nanometers, but it has the opposite effect on the measurement. These last two compressions nearly cancel, so only the compression at the fiber-to-anvil contact ought to be significant. Unfortunately, as we will see, the situation is not so simple when the micrometer is applied to thin, flexible wires or fibers.

**Fig. 3:**
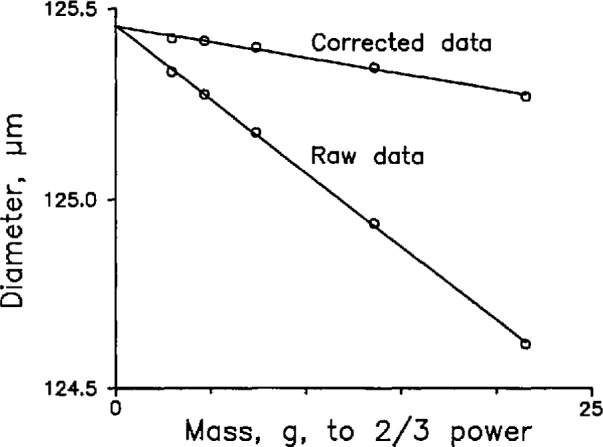
Measured diameter as a function of mass (force) to the two-thirds power. The lines are least-squares lines of best fit. The slope of the line marked “Raw data” is used to determine the diameter of the fiber in the absence of force.

To test the formulas, we measured the diameter of different fibers and steel thread wires as a function of the force the spindle exerts on the fiber. If the formula is correct, the measured diameter will be a constant, independent of force. The formula for a cylinder-to-cylinder contact shows that the compression increases in proportion to the 2/3 power of the force, so we plotted both “raw” and “corrected” diameters as a function of the 2/3 power of the mass hanging from the thread and calculated least-squares lines of best fit. Figure 3 shows the results for one of the fibers: The corrected diameter is not independent of the force (or mass); that is, the line labeled “corrected” is not horizontal. The functional dependence is apparently correct, however, because the raw data lie on a line.

The *y*-intercept is presumably the correct value; therefore, the formalism of Puttock and Thwaite effected an undercorrection up to several tenths micrometer, depending on the force. This was so for both fibers and thread wires; as far as we know, [2] is the first published report of this result. Since ferrules are often characterized with the aid of steel thread wires, the result may be important to the measurement of the inside diameters of ferrules as well.

Our colleagues in the Precision Engineering Division have proposed the following explanation for the formulas’ apparent breakdown: Thin steel wires or optical fibers are flexible. When they are pressed against the cylindrical anvil, they bend slightly, as if to wrap around the anvil. As a result, the pressure of the flat spindle against the fiber is greatest at the center of the spindle, directly opposite the point of contact between the fiber and the anvil. This pressure decreases in both directions away from the center of the spindle; as a result, the contact between the fiber and the spindle is not the uniform line contact assumed by the formulas. Since there is greater pressure on the fiber near the center of the spindle, the effective area of contact is reduced, and the deformation is greater than what is predicted by the formula.

Besides the fact that the formulas always under-correct (never overcorrect), we have three bits of evidence in favor of this explanation. First, the Precision Engineering Division tested the formulas on steel wires that had increasing thickness and therefore increasing stiffness; the prediction of the formula improved as the thickness of the wire increased. Second, silica fibers are more flexible than steel thread wires of the same thickness, and we have found that the formulas are poorer predictors of the deformation of silica fibers than of steel wires. Finally, when we replace the steel spindle with a silica spindle and prepare a graph like Fig. 3, we find that the slope of the raw data line is different than when we use the steel spindle. This suggests that at least part of the discrepancy occurs at the spindle-to-fiber contact.

### 2.3 Calculation of Diameter and Its Uncertainty

We have made no attempt to calculate the deformation of the fiber-to-spindle contact. Rather, we use the measured slope of the line labeled “Raw data” in Fig. 3 to extrapolate from the measured diameter to the true diameter, or the diameter in the absence of deformation.

**Fig. 4:**
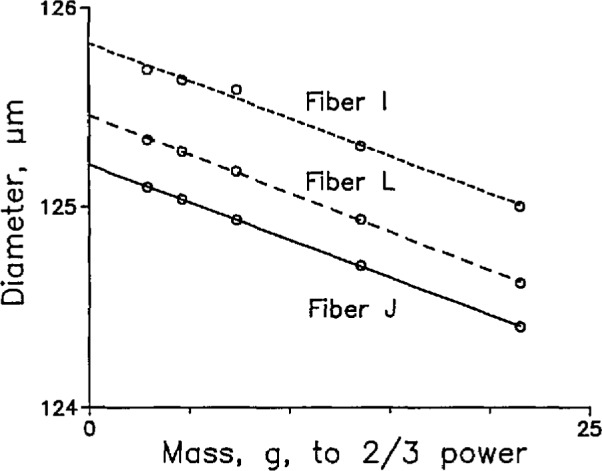
Raw data for specimens of three different fibers as a function of mass and showing that the slope is invariant.

Because all the fibers we are interested in have approximately the same diameters and are made of the same materials, the slopes of the lines of best fit are in principle the same. Figure 4 shows raw or uncorrected data similar to those of Fig. 3. Fibers I, L, and J are different fibers made by different manufacturers. The slopes of the lines of best fit are, within statistical uncertainty, the same.

We can therefore use any mass to measure the diameter and then extrapolate to zero mass by using the measured slope instead of the formulas. Table 1 shows the results of measurements on six specimens of the same three fibers as those labeled in Fig. 4. Uncertainties are calculated values, expressed as one standard deviation [10]. The overall uncertainty of the slope is given by the root-sum-of-squares (rss) average (0.0011) of the individual uncertainties divided by $\sqrt{6}$, or 0.0004. The *y*-intercept is not stated in the table because it is different for each specimen.

We chose a mass of 20 g because, with that mass, the deformation of the fiber is not great and there is little danger of damaging the spindle or the anvil, yet the force is enough to make a solid contact without causing the spindle to bounce. We used the slope given in Table 1 to extrapolate to the diameter of the fiber with zero mass (force). The statistical uncertainty of measuring that slope gives rise to a systematic uncertainty of the diameter of about 3 nm (1*σ*). Additionally, the random uncertainty of a single set of measurements is typically about 8 nm. An uncertainty of 8 nm results from the roughness of the anvil; 10 nm from the possibility of a burr on the anvil; and an estimated 10 nm from taper or internal stress in the fiber (Sec. 5). A systematic uncertainty of our measurement of the force equivalent to 1/2 g gives rise to an uncertainty of 10 nm. Other uncertainties we have been able to identify are less than 2 nm (Table 2).

We follow the ISO formalism for propagating uncertainties and assume that measured (Type A) uncertainties display a Gaussian probability distribution, whereas inferred (Type B) errors display a rectangular probability distribution [11]. We combine uncertainties by adding their variances in quadrature (where the variance of a rectangular probability distribution is one-third the square of its half-width). In this way, we arrive at an estimated standard deviation of about 14 nm. We take the overall uncertainty of any diameter measurement to be ±3 times this value, or ±45 nm.

### 2.4 Control Chart

**Fig. 5:**
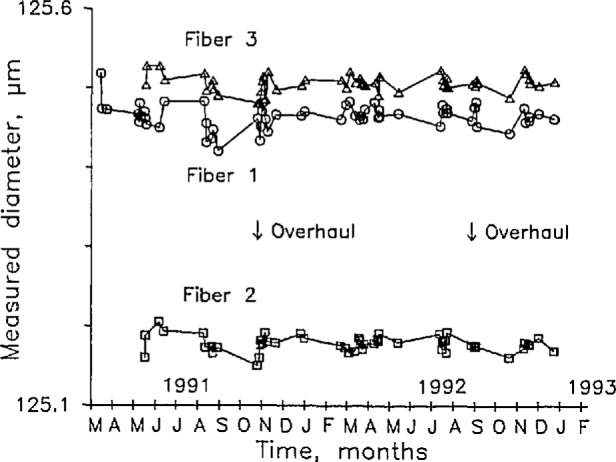
A control chart for the contact micrometer: the measured diameter of three fiber ends as a function of time for nearly 2 years that began in March 1991. The micrometer was overhauled in October 1991, and August 1992.

We prepared three fibers in retractable holders that allow us to preserve the ends indefinitely. We are therefore able to measure the diameter of the same specimens repeatedly in order to estimate stationarity and long-term drift. Figure 5 is a control chart, or a graph of measured diameter as a function of time, for nearly 2 years, beginning in March 1991, and continuing through January 1993.

From May through September 1991, the chart suggested a slight downward drift, though that drift was largely masked by the day-to-day uncertainty of the measurements. We attributed the drift to friction in the air bearing. We disassembled the bearing and cleaned it, and realigned and recalibrated the micrometer. In addition, the spindle is attached to an air supply by a soft plastic tube that can put a slight force on it. We balanced the spindle very carefully by adjusting the tube until the balance point of the spindle was just in front of the anvil; only then did we attach the mass to the spindle. This is a more accurate way of balancing the spindle than we had been using before the overhaul. Since the overhaul in late October 1991, we see no evidence of systematic changes in the control chart.

## 3. Confocal Microscope

We built the scanning confocal microscope (SCM) because it permits direct inspection of the fiber endface, whereas most other systems operate at some point along the length of the fiber [2,12,13]. The light in the SCM is spatially and temporally coherent (a single-mode laser is used for illumination), so the image does not suffer from the systematic errors associated with partially coherent illumination [7]. For this reason, the SCM is more accurate than conventional microscopy or gray scale analysis, both of which use partially coherent illumination.

We designed the SCM to measure optical fiber diameters specifically, but we think the system has the potential to be applied to many objects of interest in video microscopy and critical dimensional measurements. In particular, because out-of-focus object points are invisible, the SCM is preferable to a conventional microscope for measurements of objects, such as ridge waveguides or features on integrated circuits, whose height exceeds the depth of field of the instrument [3], or a fraction of one micrometer.

**Fig. 6:**
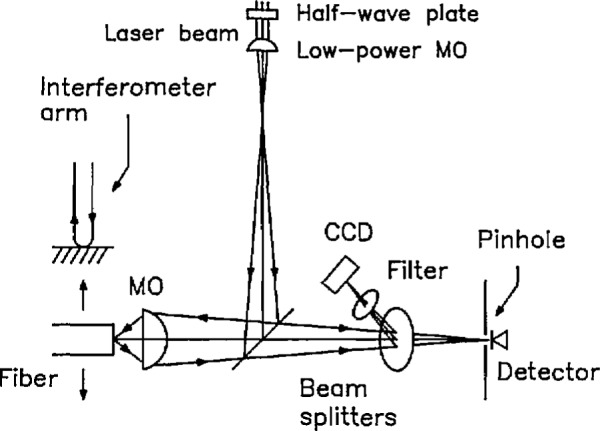
A schematic drawing of the scanning confocal microscope. The fiber is scanned vertically and its position is monitored interferometrically.

Figure 6 is a schematic drawing of the SCM, which is discussed in more detail in [3] and [4]. The object, a fiber or a linewidth standard, for example, is mounted on a three-axis translator that has a nominal step size of 0.1 µm. The object is scanned vertically under computer control while the confocal microscope, illuminating laser, and detector remain stationary on the table. The position of the object is measured with a commercial interferometer, as above (Sec. 2).

To illuminate the specimen, we use a linearly polarized single-mode He-Ne laser with a wavelength of 633 nm. The beam is passed through a half-wave plate so that the plane of polarization can be rotated as necessary. The beam is chopped for phase-sensitive detection. It is focused to a point with a low-power microscope objective and then expands and reflects from a beam splitter. The beam that reflects from the beam splitter is focused by a 40 ×, 0.65 NA objective onto the object. That objective also focuses the beam reflected from the object onto a pinhole that precedes a detector. The pinhole is located so that the objective operates at the proper magnification (proper tube length) and is the same optical distance from the beam splitter as is the focal point of the low-power objective.

The beam reflected from the object is reflected by a second beam splitter onto a video camera that allows inspecting the quality of the fiber endface and locating the image point with respect to the scanning pinhole. The second beam splitter reflects the beam out of the plane of the page in order to help cancel the astigmatism of the first. This is necessary because we did not design the instrument with infinity-corrected objective lenses, or lenses whose long conjugate is infinite.

We position the specimen near the focal point of the objective by backlighting it with a ring illuminator (not shown) and exciting the core of the fiber by illuminating the far end. We then use the video monitor to superimpose the reflected laser light onto the core of the fiber. The filter that precedes the monitor has a central wavelength of about 550 nm and a passband of about 100 nm. By eliminating infrared radiation from the source, this filter reduces chromatic aberration and ensures that the light from the white-light source and the laser light are parfocal. It also protects the video monitor from overexposure to the laser light.

We perform fine focusing manually by maximizing the photocurrent from the detector. To ensure sharp edge responses, the specimen is refocused near each edge just before the measurement scan across that edge. A scan across the fiber diameter takes approximately 1 min.

A silicon photodetector with a built-in operational amplifier is located behind the pinhole and measures the reflected light intensity. The output of the detector is synchronously detected by a lock-in amplifier. Data are obtained by moving the stepping motors in 0.1 µm steps. These stepping motors are controlled by a feedback loop that uses a linear encoder; the loop is evidently slightly underdamped, and the motors overshoot and then drift back and forth about 50 nm with a period of a few tens of seconds. The instrument is within specification, but the overshoot and drift cause noisy data near the edge. We could find no way to change the damping of the loop. If, however, we use the software to turn off the motors a specific time (about 0.1 s) after the command to step has been issued, we obtain stable data. Since we measure position with the interferometer, we have no need to rely on the encoder for position and would have been better off with a system that did not have a feedback loop.

After the stepping motor has completed its motion, we wait four time constants (0.4 s, altogether) of the lock-in amplifier before recording the detector output. The interferometer then determines the vertical position of the fiber by averaging three readings.

### 3.1 Theory

In [4], Mechels and Young showed that, in scalar theory, the geometrical image of an isolated dielectric edge is located at the inflection point of the electric field distribution in the vicinity of the edge. They used the inflection point of the electric field, rather than the point whose intensity is one-quarter of the peak intensity, to locate an edge because it is convenient, requires slightly less data acquisition, and is not affected if the reflectance of the specimen is not uniform.

They further showed experimentally that the inflection point can also be used to locate a metallic edge deposited on a glass substrate, provided that the light was polarized with its electric field vector parallel to the edge. They suggested that this was so because that polarization adheres to the same boundary conditions as a scalar wave, whereas the opposite polarization does not. Since then, we have calculated diffraction patterns of an edge as a function of polarization and distance from the edge. The two polarizations converge to the same diffraction pattern within a few hundred wavelengths of the plane of the edge, or far less than the distance between the edge and the aperture stop of our microscope objective. We therefore think that the polarization dependence of our measurements may have been the result of the oxide anti-reflection coating on the chromium lines, rather than of the boundary conditions at a metal edge. We plan to perform similar measurements with gold lines that are not coated with an oxide; preliminary results indicate that the measured widths of such lines either are independent of polarization or are more nearly so than the measured widths of the oxide-coated chromium lines.

We are interested primarily in the edges of an optical fiber, where there are neither standing waves nor phase shifts as might be caused by the reflection from a glass substrate. Further, the impulse response of the SCM is never less than 0 [14]. For these reasons, the edge response displays no zero crossings, there is no ambiguity in taking the square root of the measured data, and the electric field amplitude in the neighborhood of the edge is just the square root of the measured intensity. When examining a chromium-on-glass target, we contacted a cover slip to the substrate with an index-matching oil. (We use a microscope objective that is corrected for the spherical aberration of the cover slip and suspend the cover slip in air when we examine an object that is not contacted to a cover slip.) The index-matching fluid ensures that the reflectance from the glass substrate is very nearly 0. The same arguments hold, and we are able to calculate the edge response by calculating the square root of the data, as with a fiber.

### 3.2 Detector Aperture

In principle, the detector in the SCM should have a vanishingly small diameter [15]. More practically, we have used the sampling theorem to estimate that its *diameter* should be less than one-half the Airy disk *radius.* Using a larger diameter is mathematically equivalent to partially coherent imaging and guarantees a systematic error that we estimated in [4] to be a few tens of nanometers. Here we show experimental data regarding this systematic error.

We used the four widest lines on Standard Reference Material 475. This is a series of chromium lines deposited on a glass substrate; their widths have been measured by our laboratory in Gaithersburg with a total uncertainty of 60 nm. For this work, we used the parallel polarization only.

**Fig. 7:**
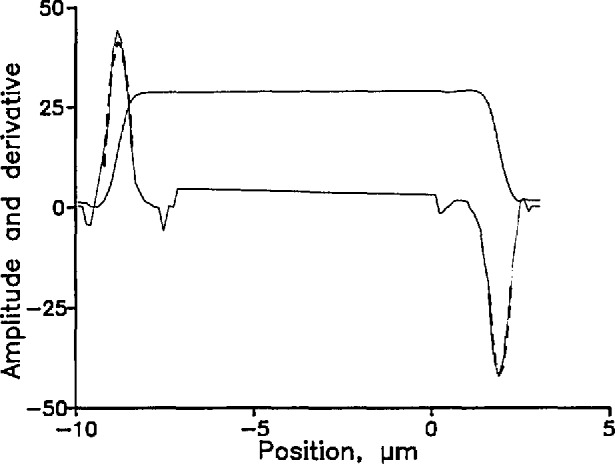
Electric field amplitude (square root of the intensity) and its derivative. Sean of a 10.71 ±0.06 µm ehromium-on-glass line from SRM-475. The dashed curves are a nine-point parabolic fit to the derivative in the region of the edges.

Figure 7 shows the electric-field amplitude (square root of the intensity) as a function of position for a 10.71 µm line. To save time, we take data only in the neighborhood of the edges. The curve is numerically differentiated, and parabolas (dashed curves) are fitted to the derivatives in the neighborhood of the peaks. The purpose of fitting the parabolas is both to smooth the data and to interpolate between pixels. The peaks of the parabolas are approximations to the true inflection points. The distance between the inflection points of the two edges is the width of the object.

**Fig. 8:**
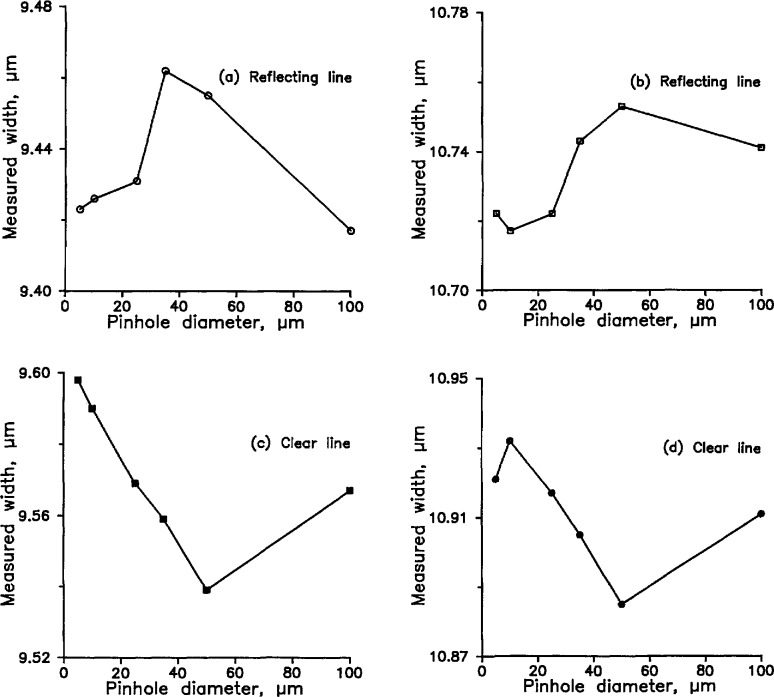
(**a**–**d**). Widths of four SRM lines measured by the SCM and plotted as a function of the diameter of the pinhole in front of the detector.

We measured the widths of each of the four lines with a variety of pinholes in front of the detector. To reduce the effect of detector nonlinearity, we adjusted the intensity of the incident light so that the peak photocurrent was approximately the same in all the measurements. The results are shown in Fig. 8 (a–d), which plots measured width as a function of pinhole diameter for both reflecting lines and clear lines on a reflecting background. The Airy disk radius, for comparison, is about 24 µm.

**Fig. 9:**
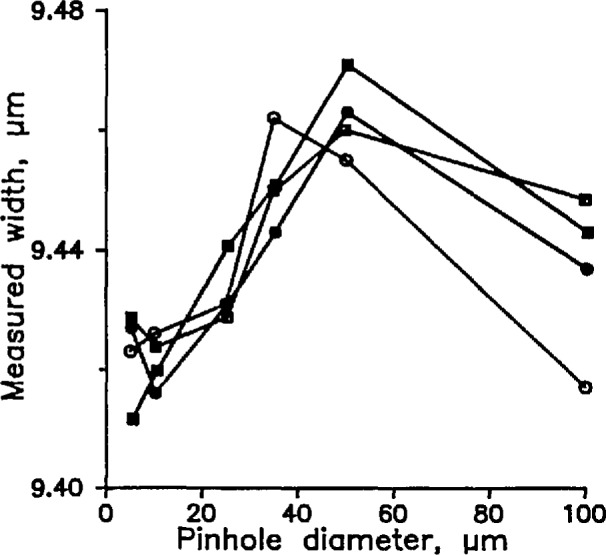
A composite of the data of Fig. 8 superimposed on the axes of Fig. 8(a). Figures 8(e) and (d) are inverted. The vertical scale has no significance.

Figure 9 is a composite of the four curves of Fig. 8 superimposed on Fig. 8 (a). Two of the curves have been inverted, and the vertical positions of the curves have been adjusted by eye for a sort of best fit. The data show a maximum systematic error of perhaps 40 nm with the 50 µm pinhole. As the pinhole radius approaches 0, random uncertainty obscures whether the curves approach an asymptote or not, but it is safe to say that using the 10 µm pinhole will result in a systematic error no larger than 5 or 10 nm. This systematic error will be positive for reflecting lines and negative for clear lines on a reflecting background. It is a curious quirk, incidentally, that the systematic error decreases somewhat when the pinhole diameter is increased to 100 µm. We do not know whether the systematic error would behave the same way if we had used the 25%-intensity criterion instead of the inflection point.

### 3.3 Linewidth Measurement

The 10 µm pinhole gave a substantially higher signal-to-noise ratio than the 5 µm pinhole, so we adopted 10 µm for subsequent measurements. Table 3 compares our measured data with Gaithersburg’s results, which we call the canonical values. Our values agree well with the canonical values but show a possible systematic uncertainty whose sign depends on whether the line is clear or reflecting.

### 3.4 Fiber Diameter

**Fig. 10:**
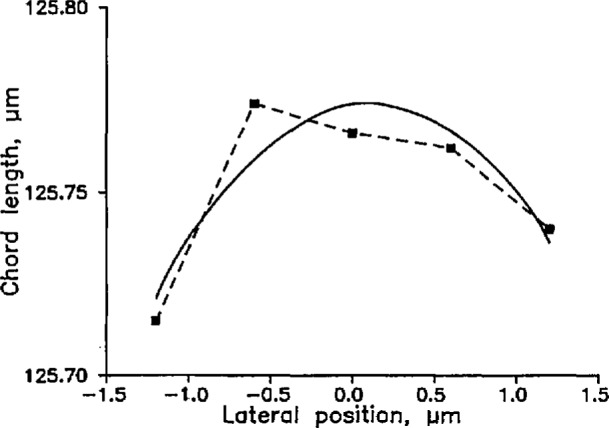
Fiber diameter measured by the SCM as a function of lateral position. The peak of the best-fit parabola is assumed to give the true diameter.

To measure fiber diameters, we first estimated the location of the center of the cladding by finding the center of the core. Then we scanned five chords in a raster that surrounded the core center and measured the lengths of these chords. The chords were 0.6 µm apart, and, at most, only one of the chords is the diameter we seek. To estimate the true diameter, therefore, we plotted chord length as a function of the perpendicular distance from the core center and fitted the data to a parabola (Fig. 10). The peak of the parabola is the measured diameter. The random uncertainty of these measurements is approximately 40 nm (3*σ*); we discuss the systematic uncertainty below (Sec. 5).

## 4. Interference Microscope

We also constructed a white-light interference microscope which uses a Mirau interference objective [16] and a partial contact method for locating the fiber surfaces [5]. We chose a Mirau objective over the Michelson objective used in previous works [17,18] because of its stability, ease of operation, and higher magnification. We chose a partial contact method because, otherwise, we would have to know the index profile and material dispersion of a test fiber for absolute measurements [18].

**Fig. 11:**
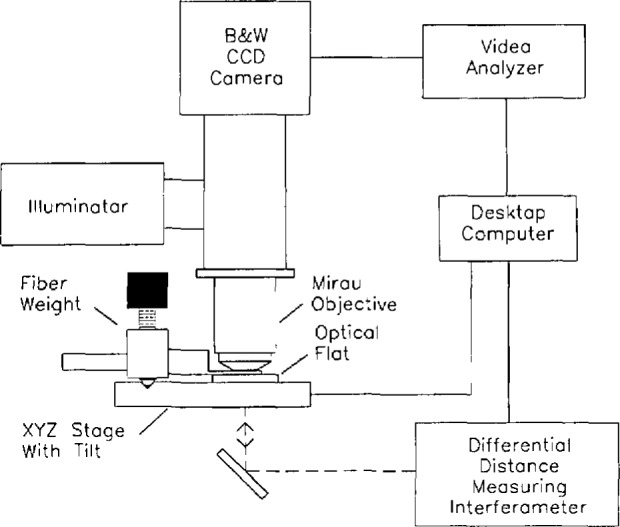
A white-light interference microscope system based on Mirau objective.

We constructed the system from commercial metallurgical microscope parts; it uses bright-field illumination from a halogen lamp, a binocular eyepiece, and a CCD array video camera with 400 × overall magnification (Fig. 11). The camera is connected to a video analyzer and monitor. An optical flat is held perpendicular to the optical axis with a high quality mirror mount on a precise three-axis translation stage. The position of the flat is monitored by a commercial interferometer with a least count of 1.25 nm. The interferometer, translation stage, and video analyzer are all controlled by computer, and repeated measurements can be made automatically.

The mirror mount has been modified to hold a fiber holder along with a cantilevered brass weight which holds the fiber against the optical flat. The fiber holder consists of a stainless steel vee groove, and the fiber is held into the groove by a wedge of silicone rubber. The holder fits into a fixture that positions the fiber on top of the flat. It can be interchanged among all three of our instruments. Before making a measurement, the mirror is aligned with the axis of the microscope by observing uniformly colored fringes from the flat across the entire field of view. When the eyepiece is used to observe the colored fringes, alignment within ±75 µrad can be achieved.

**Fig. 12:**
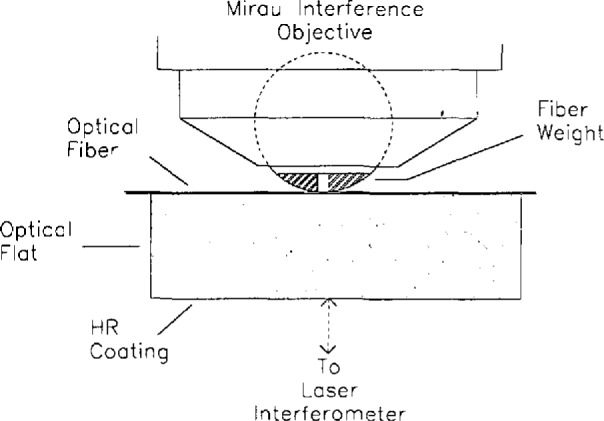
A schematic drawing of the fiber held in contact with the optical flat.

The brass weight has a semi-circular cross section and is thin enough to fit between the fiber and objective with the curved side toward the fiber. It has a 0.76 mm slot through which the fiber and flat are viewed (Fig. 12). The weight can be oriented using two precise 1/4-80 screws so that even pressure is applied to the fiber from both contact points. The weight deforms the fiber by about 0.5 µm, but the deformation is localized to a region within 20 µm of the edge of the slot. Measurements of the fiber diameter are made 380 µm from both fiber-weight contacts.

Contact between fiber and flat is verified within 50 nm by viewing white-light interference fringes analogous to Newton’s rings but linear because the fiber is cylindrical. To observe these fringes, the microscope is focused approximately 100 µm below the surface of the flat. The video analyzer verifies that the central dark fringe is as dark as the next outermost fringe, and all fringes are parallel and colorless. When slight pressure is applied to the brass weight, the central portion of the fiber rises away from the surface and the central fringe becomes hourglass-shaped. If one end of the fiber rises, the pressure is not uniform, and the weight needs to be rotated about its cylindrical axis using the precise screws.

Fringes from the top of the fiber should be colored uniformly along the length of the fiber and parallel (provided that the fiber diameter is uniform). Particulates larger than 25 nm in diameter on or adjacent to the fiber can be detected by a variation of the color of the fringes. Cleaning the fiber using spectroscopic grade methanol in an ultrasonic cleaner is effective in removing stray dirt. The flat is cleaned with spectroscopic grade methanol using the “drop-and-drag” method commonly used to clean optics with fragile coatings.

When the fiber is contacted to the flat and even pressure is applied, the fiber appears to wring onto the flat; that is, it makes optical contact and is held to the flat by a van der Waals force, which has a range of a few nanometers. We therefore think that the fiber is in intimate contact with the flat and that measurement of the distance between the flat and the top of the fiber is equivalent to measuring the diameter of the fiber with very small error; see the discussion of replacement uncertainty, below.

**Fig. 13:**
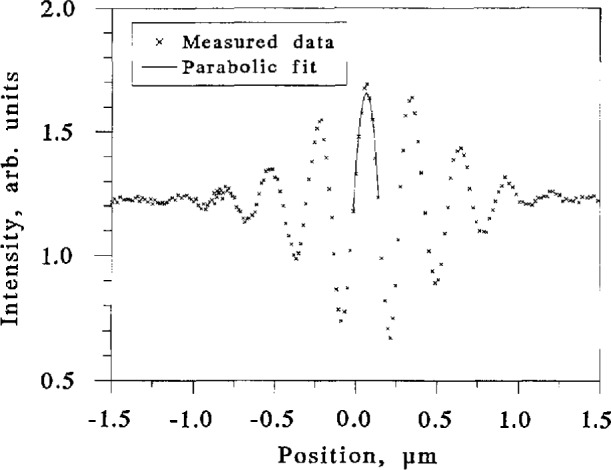
A white-light interferogram with parabolic fit to central fringe.

The top surface of the fiber or flat is located by scanning the stage and using the video analyzer to record the intensity of the white-light fringes while the position of the stage is monitored. The central fringe of the interferogram is then fitted to a parabola to average out random fluctuations in intensity and position tracking (Fig. 13). The region of the video image to be sampled is located using a set of cross hairs on the video monitor. Since the field of view is a few fiber diameters in extent, lateral motion of the translation stage is not required during a measurement. To measure the diameter of a fiber, the flat and the top of the fiber are located, and the distance between them is calculated. The flat is then relocated and the difference is again calculated. The average of these two measurements is one datum which has been corrected for linear drift. When this measurement is repeated without fiber replacement, the uncertainty of the measured mean diameter is about 3 nm (1*σ*). When the fiber is removed and replaced between measurements the uncertainty increases to 8 nm. The additional uncertainty may arise because the glass surface is rough (on a nanometer scale) and we cannot accurately reposition the fiber on the flat. That the uncertainty is only 8 nm is consistent with our assumption that the fiber is held to the flat by a van der Waals force.

The accuracy with which the cross hairs can be placed on top of the fiber is limited by quantization noise as well as random uncertainties. Slight offset of the cross hair makes the measured fiber diameter too small. To estimate the severity of the problem, we replaced the 20 × Mirau objective with a 40 × Mirau objective. We made six measurements on a control fiber and alternated them with measurements made with the 20 × objective. The 40 × objective gave values 6±4 nm (1*σ*) larger than the 20 × objective. If the probability distributions for placement of the cross hair are the same for both objectives, the mean offset of the measured diameter using the 40 × objective is four times smaller than the offset for the 20 × objective. That is, we require an additive correction of (4/3) × 6 nm, or 9±5 nm. All diameters quoted in this paper include this additive correction and its uncertainty. The overall uncertainty of 30 nm (3*σ*) is calculated by adding the uncertainty of 5 nm and the replacement uncertainty in quadrature.

## 5. Results

**Fig. 14:**
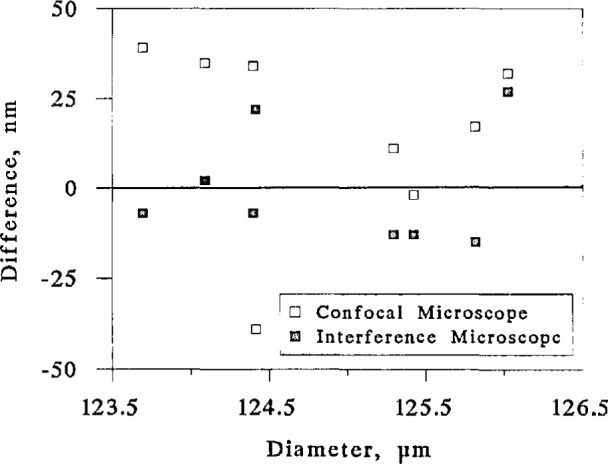
Comparison of measurements by scanning eonfoeal mi-crosecpe and white-light interference microscope with measurements by contact micrometer. The micrometer measurements are represented by the horizontal line.

We measured the diameters of several fibers with all three instruments. We took care that each set of measurements was made by a different operator and that the operators did not discuss their results until all measurements were completed. We measured two diameters of each of four fibers, for a total of eight diameters. The results are plotted in Fig. 14 as the arithmetic difference between measurements made by each of the two microscopes and those made by the micrometer as a function of the diameter measured by the micrometer. That is, the horizontal line represents the micrometer measurements, and the points represent differences from those measurements. The open squares are the measurements made by the confocal microscope, and the solid squares, by the interference microscope.

All three sets of measurements agree within their estimated experimental uncertainties. The arithmetic average of the confocal microscope measurements, however, exceeds the micrometer measurements by 16 nm, and the rss average exceeds the micrometer measurements by 29 nm. If there were a large systematic difference between the two instruments, then all the data points in Fig. 14 would have the same sign, and the arithmetic average would roughly equal the rss average. Similarly, if the arithmetic average were much less than the rss average, we could rule out a significant systematic difference between the two instruments. The arithmetic average of 16 nm is only slightly less than the rss average and is very roughly the increment by which the confocal microscope measurements differed from the Gaithersburg measurements of the metallic lines. Part of this discrepancy could be due to the finite width of the pinhole. Beyond that, we cannot explain it and do not know whether it represents a systematic error in the confocal microscope.

The single outlying point near the bottom of the graph in Fig. 14 is a measurement taken on a fiber that we think had very high stress and may have become deformed when it was cleaved; see below, at the end of this section. Without the outlying point, the arithmetic average difference between the two sets of measurements would be 24 nm, not 16, and the rss average difference would be 28 nm. The case that there is a real systematic difference between the two instruments could therefore be made more convincingly.

We used the interference microscope to measure the diameters of the same set of fibers. We had to remove the fibers from their holders and remount them, so we remeasured their diameters with the micrometer, in case there had been rotation when the fibers were remounted. (This is the reason that we show the data in relation to the micrometer, rather than present all three datasets in Fig. 14.) We show the results as the solid squares in Fig. 14. The arithmetic average difference between the two datasets is −1 nm, and the rss average is 15 nm. The agreement between the micrometer and the interference microscope is well within experimental error.

Finally, to ensure that fiber ends are not deformed by cleaving, we used the interference microscope with the 40 × objective lens to visually examine a number of cleaved fibers, including the outlier in Fig. 14, at their ends. We could find no evidence for any deformation. Further, finite-element analyses of fibers with realistic axial stress distributions with magnitudes in the range of 20–50 MPa yielded radial deformations of a few nanometers at the end of the fiber. Since our SRM is a single-mode fiber with a homogeneous cladding and a low drawing tension, we do not think that internal stress is a factor. We caution, however, that certain high-stress fibers, such as polarization-maintaining fibers, might display enough internal stress that a measurement of a cleaved end will not be representative of the diameter of the fiber far from the end; this is a topic that may bear further investigation.

## 6. Discussion

We estimate the overall uncertainty (3 standard deviations of the mean) of the micrometer as 45 nm; of the confocal microscope as 40 nm; and of the white-light interference microscope as 30 nm. The interference microscope and the micrometer agree remarkably well, whereas the confocal microscope may display an additional systematic error of the order of 20 nm. The microscopes are hard to use, so we use the micrometer to characterize Standard Reference Materials that are available from our offices in Gaithersburg [19].

These SRMs consist of a carefully cleaved fiber end in a retractable holder. The diameter of the end is measured at four angles with respect to a fiducial mark on the holder. All four diameters thus determined are reported, as well as their average. The individual diameter measurements are specified accurate within ±42 nm. For the convenience of the user, the SRM includes a short pigtail in case the core is to be illuminated. The SRM does not, however, include a specification of non-circularity or core-cladding decentering.

## Figures and Tables

**Table 1:** Intercept uncertainty, slope, and slope uncertainty of six specimens

Fiber	Intercept uncertainty, µm	Slope, µm/g^2/3^	Slope uncertainty, µm/g^2/3^
I	23	−0.0377	0.0019
J	2.8	−0.0375	0.0002
L	5.5	−0.0386	0.0005
I′	14.4	−0.0380	0.0012
I″	9.4	−0.0369	0.0008
L′	10.8	−0.0378	0.0009

rss mean	14	−0.0378	0.0011

**Table 2:** Tabulation of uncertainties

Source of uncertainty	Criterion	Uncertainty, nm
(a) Measured uncertainties		

Noise	Calculated standard deviation	8
Correction for deformation [1]	Standard deviation of calculated slope	3

(b) Inferred uncertainties		

Correction for deformation [1]	Error of force equivalent to ±1/2 g	10
Surface roughness	± 1/2 of correction	8
Burr on anvil	Control chart	10
Deformation or taper	Stress analysis & interference microscope	10
Cosine	0.2°	1
Abbe offset	1 µrad (0.2′) × 2 mm offset	2
Wavelength	Fluorescence linewidth	0.3
Index of refraction of air under average conditions	Uncertainty of calculation	<0.1
Variation of barometric pressure	±4 kPa	1
Variation of ambient temperature	±5 K	0.5
Variation of relative humidity	±50%	<0.1

Combined uncertainty	1*σ*	14

Expanded uncertainty	3*σ*	43

**Table 3:** Measured widths of chromium lines (SRM-475).^a^ All measurements in micrometers. Parallel polarization

	Canonical value	Measured value	Difference
Reflecting lines	9.40	9.43	0.03
	10.71	10.72	0.01
Clear lines	9.63	9.59	−0.04
	10.97	10.93	−0.04

a Random uncertainty of SCM is 3*σ* = 40 nm; total uncertainty of canonical values is 60 nm.

## References

[1] Young M, Day GW, Franzen DL (1990). Standards for optical fiber geometry measurements. Technical digest—Symposium on optical fiber measurements, 1990.

[2] Young M (1990). Fiber cladding diameter by contact micrometry, Digest, Optical fiber measurement conf.

[3] Mechels S, Young M (1992). Scanning confocal microscope for accurate dimensional measurement. Proc Soc Photo-Optical Instrum Engrs.

[4] Mechels S, Young M (1991). Scanning confocal microscope for precise measurement of optical fiber diameter. Proc Soc Photo-Optical Instrum Engrs.

[5] Hale PD, Franzen DL, Day GW, Franzen DL (1992). Fiber cladding diameter measurement by white light interference microscopy. Technical digest —Symposium on optical fiber measurements, 1992.

[6] Fiberoptics Test Procedure FOTP-176 Measurement method for optical fiber geometry by automated gray-scale analysis.

[7] Mechels S, Young M (1991). Video microscope with sub-micrometer resolution. Appl Opt.

[8] Collyer A, Raine KW, Baines JGN (1990). Investigation into the use of a scanning confocal microscope as a primary measurement method for determining optical fiber diameter. Digest, Optical fiber measurement conference.

[9] Puttock MG, Thwaite EG (1969). Elastic compression of spheres and cylinders at point and line contact.

[10] Taylor JR (1982). Introduction to error analysis.

[11] Anonymous (1992). Guide to the expression of uncertainty in measurement.

[12] O’Sullivan MS, Grandsen DW (1987). A Fizeau fringe interferometer for fiber outside diameter measurements. Proc Soc Photo-Optical Instrum Engrs.

[13] Saunders MJ, Day GW, Franzen DL (1988). Non-contact, interferometric determination of the outside diameter of optical fibers. Technical digest —Symposium on optical fiber measurements, 1988.

[14] Obarski GE, Drapela TJ, Young M Scanning confocal microscopy for measuring diameter and linewidth: numerical modelling, in OE Lase 92—Biomedical Optics/Lasers, Sensors, and Spectroscopy. Proc Soc Photo-Opt Instrum Engrs.

[15] Wilson T, Sheppard C (1984). Theory and practice of scanning optical microscopy.

[16] Komatsu H Interferometry: Principles and applications of two-beam and multiple-beam interferometry.

[17] Emig KA, Day GW, Franzen DL (1990). A comparison of interferometric techniques for fiber cladding diameter measurements. Technical digest—Symposium on optical fiber measurements, 1990.

[18] Saunders MJ, Day GW, Franzen DL (1988). Noncontact interferometric determination of the outside diameter of optical fibers. Technical digest—Symposium on optical fiber measurements, 1988.

[19] National Institute of Standards and Technology Standard Reference Material Program.

